# Unraveling the Molecular Threads: A Comprehensive Review of the Pathogenesis and Therapeutic Insights Into Allergic Rhinitis

**DOI:** 10.7759/cureus.64410

**Published:** 2024-07-12

**Authors:** Abhijeet Sharma, Prasad Deshmukh, Shraddha Jain, Sagar Gaurkar, Ayushi Sharma

**Affiliations:** 1 Otorhinolaryngology and Head and Neck Surgery, Jawaharlal Nehru Medical College, Datta Meghe Institute of Higher Education and Research, Wardha, IND; 2 Dental Sciences, People's College of Dental Science and Research Centre, Bhopal, IND

**Keywords:** personalized medicine, immunotherapy, inflammatory mediators, molecular mechanisms, pathogenesis, allergic rhinitis

## Abstract

Allergic rhinitis (AR) is a prevalent inflammatory disorder of the nasal mucosa, triggered by allergen exposure and characterized by symptoms such as sneezing, nasal congestion, itching, and rhinorrhea. This comprehensive review aims to unravel the molecular mechanisms underpinning AR, exploring the pathogenesis from allergen recognition to chronic inflammation and tissue remodelling. Central to the disease are immunoglobulin E (IgE)-mediated hypersensitivity reactions, involving key inflammatory mediators and cellular players such as mast cells, eosinophils, and T cells. Genetic predisposition and environmental factors also play significant roles in susceptibility and disease progression. Therapeutic strategies for AR are varied, ranging from symptomatic relief through antihistamines and nasal corticosteroids to more targeted approaches like allergen-specific immunotherapy. Emerging treatments focus on novel molecular pathways, with a growing emphasis on personalized medicine to optimize patient outcomes. Despite advancements, challenges remain in fully understanding the heterogeneity of AR and developing universally effective treatments. This review synthesizes current knowledge, highlighting critical insights into the molecular basis of AR and their implications for clinical practice. It underscores the need for integrated, multidisciplinary approaches to enhance therapeutic efficacy and calls for ongoing research to address unresolved questions and explore new frontiers in AR management. Through this comprehensive synthesis, the review aims to inform and inspire future research and clinical strategies, ultimately improving the quality of life for individuals affected by AR.

## Introduction and background

Allergic rhinitis (AR), commonly known as hay fever, is an inflammatory disorder of the nasal mucosa triggered by allergen exposure [1]. It is characterized by symptoms such as sneezing, nasal congestion, itching, and rhinorrhea. AR affects individuals of all ages worldwide, with a significant impact on quality of life, work productivity, and healthcare costs. According to epidemiological studies, its prevalence has been steadily increasing over the past few decades, making it a global health concern [2]. Understanding the molecular mechanisms underlying AR is crucial for developing effective therapeutic strategies. AR is a complex disorder involving interactions between environmental allergens, the immune system, and genetic factors. By unraveling the molecular pathways involved, researchers can identify novel targets for intervention, leading to improved management of symptoms and prevention of disease progression [3].

The purpose of this comprehensive review is to provide a detailed overview of the molecular threads involved in the pathogenesis of AR. By synthesizing current knowledge from basic and clinical research, we aim to elucidate the key inflammatory mediators, cellular players, and genetic determinants contributing to the development and progression of AR. Furthermore, we will discuss the implications of these findings for the development of novel therapeutic approaches and the potential for personalized medicine in the management of AR.

## Review

Pathogenesis of AR

Overview of the Immune Response in Allergic Reactions

The immune response in allergic reactions involves an abnormal reaction of the immune system to harmless substances known as allergens. This reaction initiates a cascade of events that result in symptoms such as sneezing, itching, watery eyes, and skin rash [4,5]. In susceptible individuals, the immune system can overreact upon exposure to allergens, producing antibodies called immunoglobulin E (IgE) [5]. These IgE antibodies bind to cells like basophils and mast cells, triggering the release of inflammatory chemicals such as histamine, prostaglandins, and leukotrienes [4,5]. Allergens can trigger allergic reactions when they come into contact with the skin or eyes or are inhaled, ingested, or injected [4]. The immune system identifies these allergens as invaders, leading to an exaggerated response. The immune response to allergens can cause a range of symptoms, from mild to severe, including wheezing, itching, runny nose, watery eyes, hives, and, in severe cases, anaphylaxis characterized by throat swelling, difficulty breathing, and a drop in blood pressure [4,5]. Each type of IgE antibody has a specific "radar" for a particular allergen, explaining why some individuals are allergic to specific substances while others react to multiple allergens [5]. Genetic and environmental factors play a role in the development of allergies, with a family history of allergies being a significant risk factor for allergic diseases [5,6]. 

Role of IgE-Mediated Hypersensitivity

IgE-mediated hypersensitivity is pivotal in allergic reactions, particularly AR. The immune system produces IgE antibodies in response to exposure to allergens such as pollen, dust mites, or animal dander. When an individual with AR is sensitized to an allergen, IgE antibodies bind to mast cells and basophils, priming these cells for subsequent encounters with the allergen. Upon re-exposure to the allergen, cross-linking of IgE on mast cells and basophils triggers the release of inflammatory mediators like histamine, prostaglandins, and leukotrienes. These mediators cause the characteristic symptoms of AR, including nasal congestion, sneezing, itching, and rhinorrhea. The release of these substances leads to the inflammatory cascade and tissue responses associated with allergic reactions [4,6]. Furthermore, IgE-mediated hypersensitivity plays a crucial role in activating T helper 2 (Th2) cells, which secrete cytokines like IL-4, IL-5, and IL-13. These cytokines contribute to the recruitment of inflammatory cells to the site of allergen exposure, leading to bronchial hyperreactivity, mucus secretion, and other pro-inflammatory effects. IL-4 is particularly important as it induces IgE class switching, facilitating mast cell activation and the subsequent inflammatory responses seen in AR [7].

Key Inflammatory Mediators Involved

**Figure 1:**
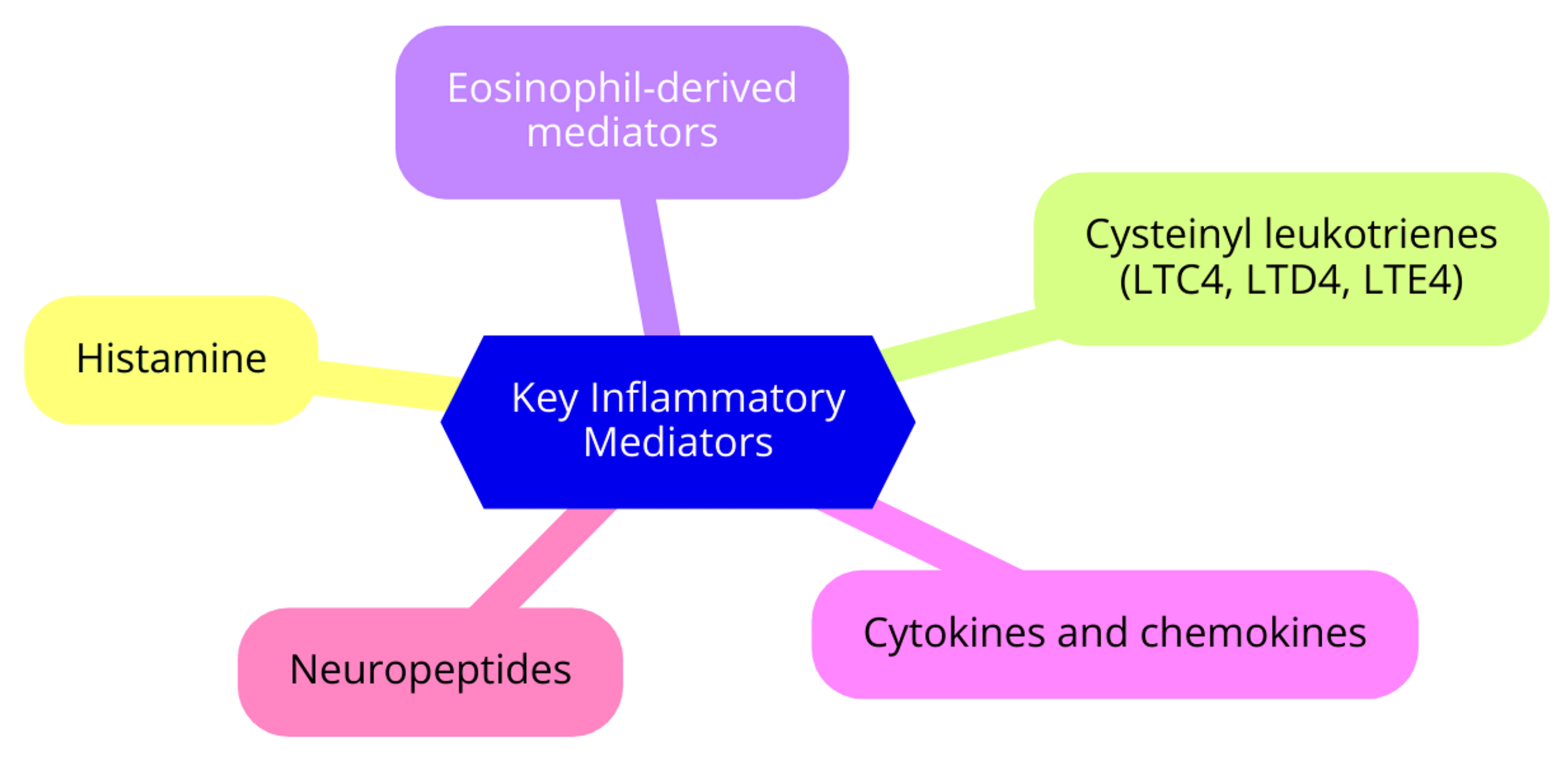
Key inflammatory mediators involved Image Credit: Dr. Abhijeet Sharma

Histamine is released by activated mast cells and basophils, inducing vasodilation, increased vascular permeability, sensory nerve stimulation, and hypersecretion from glandular cells. These actions contribute to the early-phase symptoms of AR [8,9]. Cysteinyl leukotrienes (LTC4, LTD4, LTE4) are inflammatory lipid mediators synthesized by mast cells, eosinophils, basophils, and macrophages. They induce nasal vasodilation, increase vascular permeability, and contribute to the late-phase response [8,9]. Eosinophil-derived mediators play a significant role in perpetuating the inflammatory response. Eosinophils release various pro-inflammatory mediators, including cysteinyl leukotrienes, cationic proteins, eosinophil peroxidase, and major basic proteins [8,9]. Cytokines and chemokines, such as IL-3, IL-4, IL-5, IL-13, and granulocyte-macrophage colony-stimulating factor (GM-CSF), are involved in the recruitment and activation of inflammatory cells. These pro-inflammatory molecules contribute to the chronic inflammatory state in AR [8]. Neuropeptides also contribute to the pathophysiology of AR symptoms, adding another layer to the complex network of factors involved in AR [8]. Key inflammatory mediators involved are shown in Figure 1.

Cellular Players: Mast Cells, Eosinophils, and T Cells

The cellular players involved in AR include mast cells, eosinophils, and T cells, each playing crucial roles in the pathogenesis of this condition characterized by IgE-mediated hypersensitivity reactions to environmental allergens [10-14]. Mast cells are central to the inflammatory response, releasing many inflammatory mediators upon activation. They initiate allergic responses and are often found in close proximity to eosinophils in allergic tissues. Mast cells become activated when IgE antibodies on their surface cross-link with allergens, triggering the release of histamine, tryptase, and other pro-inflammatory mediators. These substances contribute to AR symptoms such as itching, sneezing, and rhinorrhea [10-14]. Eosinophils are another type of immune cell that is significant in allergic responses. Activated by various stimuli, including allergens, eosinophils release granules containing major basic protein, eosinophil-derived neurotoxin (EDN), and eosinophil cationic protein (ECP). These mediators contribute to inflammation and tissue damage observed in AR [10-14]. Additionally, eosinophils interact with mast cells, enhancing their activation and the release of pro-inflammatory mediators [13]. T cells, particularly Th2 cells, play a role in the late-phase response of AR. Activated by allergens, Th2 cells secrete cytokines such as IL-4, IL-5, and IL-13, which promote eosinophil recruitment and activation. Th2 cells also stimulate the production of chemokines that attract eosinophils to sites of inflammation [10-14]. Furthermore, T cells interact with mast cells and eosinophils, further amplifying their activation and the inflammatory response [14].

Genetic and Environmental Factors Influencing Susceptibility

A strong family history of atopic disease stands as the most compelling evidence for a genetic influence in AR [15]. While polymorphisms of candidate genes have been linked to the clinical expression of AR, their ability to definitively distinguish an "allergic individual" from the general population remains elusive [15]. Genome-wide association studies have identified genetic variants that regulate ORMDL3 expression, contributing to childhood asthma susceptibility [16]. Twin studies indicate that genetic factors contribute more than 50% to developing allergic diseases, with heritability estimates ranging widely from 36% to 79% [17]. Environmental factors also significantly influence the onset of AR [15,17]. Air pollution, bacterial and viral infections, and lifestyle and diet changes are crucial. Epidemiological research underscores a robust association between air pollution and both the development and exacerbation of AR, with particular attention given to pollutants such as ozone, nitrogen dioxide, and particulate matter [17]. Diesel exhaust particulates can bind proteins and carry allergens, penetrating deeply into the respiratory tract [17]. Among airborne allergens, dust mites, pollens, fungi, and animal dander are extensively studied environmental factors influencing allergic conditions [17].

Molecular mechanisms underlying AR

Sensitization Phase: Allergen Recognition and IgE Production

The sensitization phase of AR involves the immune system's recognition of allergens and the subsequent production of allergen-specific IgE antibodies [18-20]. This process begins with allergen recognition by antigen-presenting cells, primarily dendritic cells (DCs), located in the nasal mucosa [18,19]. DCs express pattern recognition receptors, such as C-type lectin and Toll-like receptors, enabling them to bind and internalize allergens [18]. This leads to the activation and maturation of DCs, which then migrate to the nearby lymph nodes to present allergen-derived peptides to naive T cells [18,19]. The interaction between DCs and T cells, particularly Th2 cells, results in the differentiation and proliferation of Th2 cells [18,19]. Th2 cells subsequently assist B cells, prompting the production of allergen-specific IgE antibodies [18-20]. These IgE antibodies bind to high-affinity receptors (FcεRI) on the surfaces of mast cells and basophils, sensitizing them to the specific allergen [19]. Upon subsequent exposure to the allergen, cross-linking of IgE-FcεRI complexes on mast cells and basophils triggers the release of inflammatory mediators, including histamine, leukotrienes, and prostaglandins, which are responsible for the immediate symptoms of AR [18,19]. This sensitization phase is critical in developing AR, establishing the groundwork for subsequent allergic responses and chronic inflammation observed in the disease [18-20].

Effector Phase: Release of Inflammatory Mediators

The effector phase of the allergic response in AR is marked by the release of inflammatory mediators from various immune cells, particularly mast cells and eosinophils [8,9,21]. Upon exposure to an allergen, mast cells sensitized by IgE undergo degranulation, releasing preformed and newly synthesized mediators such as histamine, prostaglandins, and leukotrienes [8,9,21]. These mediators promptly induce symptoms like nasal itching, sneezing, and rhinorrhea in AR [8,21]. Eosinophils, predominant in the late-phase allergic response, also contribute significantly by releasing various pro-inflammatory mediators. These include cysteinyl leukotrienes, cationic proteins, eosinophil peroxidase, and major basic proteins [9]. Eosinophils additionally produce cytokines like IL-3, IL-5, GM-CSF, and IL-13, which further sustain the inflammatory process [9]. Other mediators involved in cellular chemotaxis or enhancing inflammation are either performed or rapidly synthesized from cellular components [9]. These inflammatory mediators are crucial in recruiting additional inflammatory cells such as basophils, neutrophils, T lymphocytes, and monocytes, thereby contributing to the late-phase allergic response [9].

Chronic Inflammation and Tissue Remodelling

Chronic inflammation and tissue remodelling are closely intertwined in AR, where persistent inflammation is a primary driver of structural changes in the nasal mucosa. Studies have demonstrated that AR can exacerbate the remodelling process in chronic rhinosinusitis (CRS), showing distinct impacts in CRS with nasal polyps (CRSwNP) versus CRS without nasal polyps (CRSsNP) [22]. Research has identified several key remodelling factors, including vascular endothelial growth factor (VEGF-A), collagen deposition, and matrix metalloproteinases (MMPs), which play significant roles in AR-associated tissue remodelling [22]. Increased expression levels of VEGF-A, CD31, CD34, and tissue inhibitors of metalloproteinase-1 (TIMP-1) have been observed in CRS cases accompanied by AR compared to those without AR [22]. Variations in goblet cell hyperplasia, collagen fibre density, and the expression of matrix metalloproteinase-9 (MMP-9) and transforming growth factor-beta 1 (TGF-β1) have also been noted between CRS cases with and without nasal polyps in the context of AR [22]. While some studies have not consistently detected evidence of tissue remodelling in AR, methodological challenges and environmental factors could influence assessing these structural changes [23,24].

Therapeutic approaches

Symptomatic Relief: Antihistamines, Decongestants, and Nasal Corticosteroids

Symptomatic relief for AR can be effectively managed with various medications, including antihistamines, decongestants, and nasal corticosteroids. Antihistamines block histamine from binding to cells, alleviating symptoms such as itching, sneezing, and a runny nose. They are available in multiple formulations, including pills, liquids, nasal sprays, eye drops, and skin creams. Antihistamines are particularly effective in reducing itching, sneezing, and runny nose symptoms and can be taken preemptively to provide prophylactic relief before symptoms manifest [25]. Decongestants reduce swelling in the nose's blood vessels, relieving nasal congestion and opening airways. They come in various formulations, such as nasal sprays, eye drops, tablets, capsules, liquids, and powders. Decongestants offer rapid relief from nasal congestion and can be used alongside antihistamines for enhanced efficacy. However, oral decongestants should not be used for more than a week due to the risk of rebound congestion and potential adverse effects like increased blood pressure and heart rate [26]. Intranasal corticosteroids are another essential treatment option for AR, working to decrease inflammation and swelling in the nasal mucosa. They are available as sprays and are considered the most effective treatment for persistent symptoms, offering long-term relief with fewer adverse effects compared to antihistamines and decongestants. Intranasal corticosteroids should be used under medical supervision, especially in children and pregnant women, to ensure appropriate management [27]. Combining antihistamines and decongestants can synergistically relieve symptoms, particularly nasal congestion. Over-the-counter combination products like Zyrtec-D (cetirizine and pseudoephedrine) and Claritin-D (loratadine and pseudoephedrine) are available. It's important to note that antihistamines may cause drowsiness and are not recommended for children under six without medical guidance [27].

Immunomodulatory Therapies: Allergen-Specific Immunotherapy (AIT)

AIT can modify the underlying allergic mechanism by inducing desensitization and tolerance to specific allergens [28-31]. This therapeutic approach has been utilized for over a century to desensitize individuals suffering from allergic conditions such as rhinitis, asthma, and venom hypersensitivity [29,31]. The primary mechanisms of AIT involve inducing peripheral T-cell tolerance and promoting regulatory T cells (Tregs), including FOXP3+ Tregs and IL-10/TGF-β-producing type 1 Tregs (Tr1 cells) [31]. These Tregs play a crucial role in suppressing pro-inflammatory cells like eosinophils, mast cells, and basophils while promoting the production of non-inflammatory antibodies such as IgG4 [31]. AIT can be administered through subcutaneous immunotherapy (SCIT) or sublingual immunotherapy (SLIT) routes [29,31]. SLIT is generally considered safer than SCIT, with a reduced risk of systemic reactions [29,31], although SCIT may offer greater effectiveness for certain patients [29]. Emerging immunomodulatory approaches include anti-IgE monoclonal antibodies, tryptase inhibitors, phosphodiesterase-4 inhibitors, and chemokine inhibitors, each targeting different steps in the allergic inflammatory cascade [28,30]. Despite its disease-modifying potential, AIT has limitations such as long treatment durations, high costs, reduced patient compliance, and the risk of adverse reactions [28,30]. Ongoing research aims to develop safer and more effective AIT vaccines and delivery methods to address these challenges [28,30].

Emerging Treatments Targeting Novel Pathways

AR is a complex inflammatory condition characterized by an IgE-mediated hypersensitivity reaction to environmental allergens. The pathogenesis involves a series of events triggered by allergen exposure, which activates mast cells, eosinophils, and Th2 cells. These cells release inflammatory mediators and cytokines, initiating nasal symptoms [32]. The process begins with allergen exposure and sensitization, where antigen-presenting cells, T cells, and B cells collaborate to produce allergen-specific IgE antibodies. Upon subsequent allergen exposure, IgE cross-linking on mast cells releases histamine and other mediators, causing immediate nasal symptoms. This is followed by a late-phase response characterized by the influx of Th2 cells, eosinophils, and basophils into the nasal mucosa [33]. Leukotrienes and prostaglandins also play pivotal roles in the inflammatory process, while allergen endotoxins can induce non-IgE-mediated nasal hypersensitivity through T-cell activation. In some cases, remodelling of the nasal mucosa may occur [34].

AR treatment options include allergen avoidance, pharmacotherapy (such as antihistamines, decongestants, and intranasal corticosteroids), immunotherapy, and surgical interventions. Intranasal corticosteroids are recommended as first-line pharmacological agents, with second-generation antihistamines preferred over first-generation ones [35]. Immunotherapy, administered via subcutaneous or sublingual routes, is the only treatment capable of modifying the underlying allergic mechanism by inducing desensitization and energy to specific allergens. Despite these options, many patients continue to experience persistent symptoms, underscoring the need for more effective therapies targeting AR's complex pathophysiology [36]. Emerging treatments for AR are focusing on novel pathways. Potential therapies under investigation include anti-IgE monoclonal antibodies, tryptase inhibitors, phosphodiesterase-4 inhibitors, and chemokine inhibitors. These treatments selectively target inflammatory response pathways, offering alternative options for patients with persistent symptoms [37]. Similar efforts are underway across various diseases, such as Alzheimer's, cancer, and substance use disorders. In Alzheimer's disease, research centres on neuroinflammation, microglial activation, tau aggregation, and multi-target approaches. New classes of agents targeting androgen receptor signalling, poly (ADP-ribose) polymerase (PARP), enhancer of zeste homolog 2 (EZH2), cyclin-dependent kinases 4 and 6 (CDK4/6), and immunotherapies are being explored in cancer. For substance use disorder, novel drug targets, therapeutic antibodies, and vaccines are being investigated [38,39].

Potential for Personalized Medicine Approaches

AIT is widely regarded as a personalized treatment approach because it identifies and administers specific allergens that trigger individual patient symptoms [40,41]. By leveraging biomarkers and endotyping, AIT aims to enhance treatment efficacy by precisely selecting the right patients and allergens for therapy [41]. Biologics, such as anti-IgE monoclonal antibodies, represent another personalized strategy by targeting the underlying IgE-mediated pathogenesis specific to each patient's allergic profile [37,40]. Recent phase II trials have shown promising outcomes in reducing AR symptoms, highlighting their potential benefit [37]. Emerging therapies involving small molecules are also exploring personalized approaches. These therapies target pathways like leukotrienes, phosphodiesterases, and chemokines, crucial in AR's complex pathogenesis [37,40]. Such targeted treatments promise personalized options tailored to individual patient needs. Pharmacogenomics identifies genetic and epigenetic factors influencing how patients respond to medications such as antihistamines and corticosteroids. This personalized approach aims to optimize pharmacotherapy by tailoring treatment regimens based on genetic profiles [41,42]. Furthermore, using biomarkers and endotyping techniques allows for a deeper understanding of the immunological and inflammatory mechanisms driving AR in individual patients. This knowledge facilitates personalized treatment selection and ongoing monitoring to ensure optimal therapeutic outcomes [41,42].

Challenges and future directions

Current therapies across various diseases and conditions face limitations that impact their effectiveness and patient outcomes. In cardiovascular medicine, treatments for heart failure, while advancing, still show limited efficacy in reducing mortality rates. Additionally, the high cost of medications and the logistical challenges of accessing treatment can create barriers to care. Neurological disorders like Parkinson's disease encounter challenges with current therapies, as medications such as L-dopa may not comprehensively manage all symptoms and can lead to significant side effects over time. These treatments often do not halt disease progression, leaving patients vulnerable to developing debilitating symptoms [43]. In asthma management, the effectiveness of current treatments, such as inhaled corticosteroids, in controlling symptoms and reducing mortality rates is constrained. Financial burdens associated with asthma medications further complicate patient access to treatment. Moreover, adverse effects like osteoporotic fractures and arrhythmias add complexity to managing chronic conditions like asthma. Precision targeting in research and treatment planning is crucial to developing cost-effective interventions that cater to the diverse patient needs across different regions [44]. Environmental factors significantly influence allergies, emphasizing the need to focus on environmental sustainability and healthier living conditions to mitigate allergic reactions. Prevention strategies, particularly early-life allergen exposure and specific dietary approaches, hold promise in allergy prevention. However, managing challenges in food allergy, biologics, and AIT remains unmet, underscoring the importance of ongoing research and innovation in these domains. Technological advancements in immune monitoring and diagnostic tools present opportunities to enhance patient care and outcomes. Effective communication and shared decision-making between healthcare providers and patients are crucial for informed treatment choices. Addressing challenges in allergen prediction for food safety is essential for fostering healthier and more sustainable food systems [45].

## Conclusions

This comprehensive review has elucidated the intricate molecular mechanisms underlying AR, emphasizing the roles of IgE-mediated hypersensitivity, inflammatory mediators, and the involvement of various immune cells. We have also highlighted the impact of genetic and environmental factors on disease susceptibility. The insights gained from understanding these molecular pathways are pivotal for advancing therapeutic strategies. Current treatments, while effective for symptom management, have limitations that necessitate the development of novel interventions targeting specific molecular pathways. This review underscores the importance of personalized medicine approaches, leveraging individual genetic and environmental profiles to optimize treatment outcomes. Moving forward, an integrated approach that combines clinical practice, cutting-edge research, and public health initiatives is essential to mitigate the burden of AR. Through multidisciplinary collaboration and increased public awareness, we can improve prevention, diagnosis, and treatment, ultimately enhancing the quality of life for those affected by this pervasive condition.
